# Supplementary material for: Surface Microbiome Profiling of Dental Elevators Using Third‐Generation Sequencing: Implications for Infection Control in Dental Practice
Source: Microbiologyopen. 2025 Dec 8;14(6):e70178. doi: 10.1002/mbo3.70178 (PMC12685768; doi:10.1002/mbo3.70178)
Supplement: Supplementary file 1 — customer_backup. [file MBO3-14-e70178-s001.zip › customer_backup/customer_backup/function_analysis/BugBase/treat/predicted_phenotypes/treat.Forms_Biofilms.pdf]

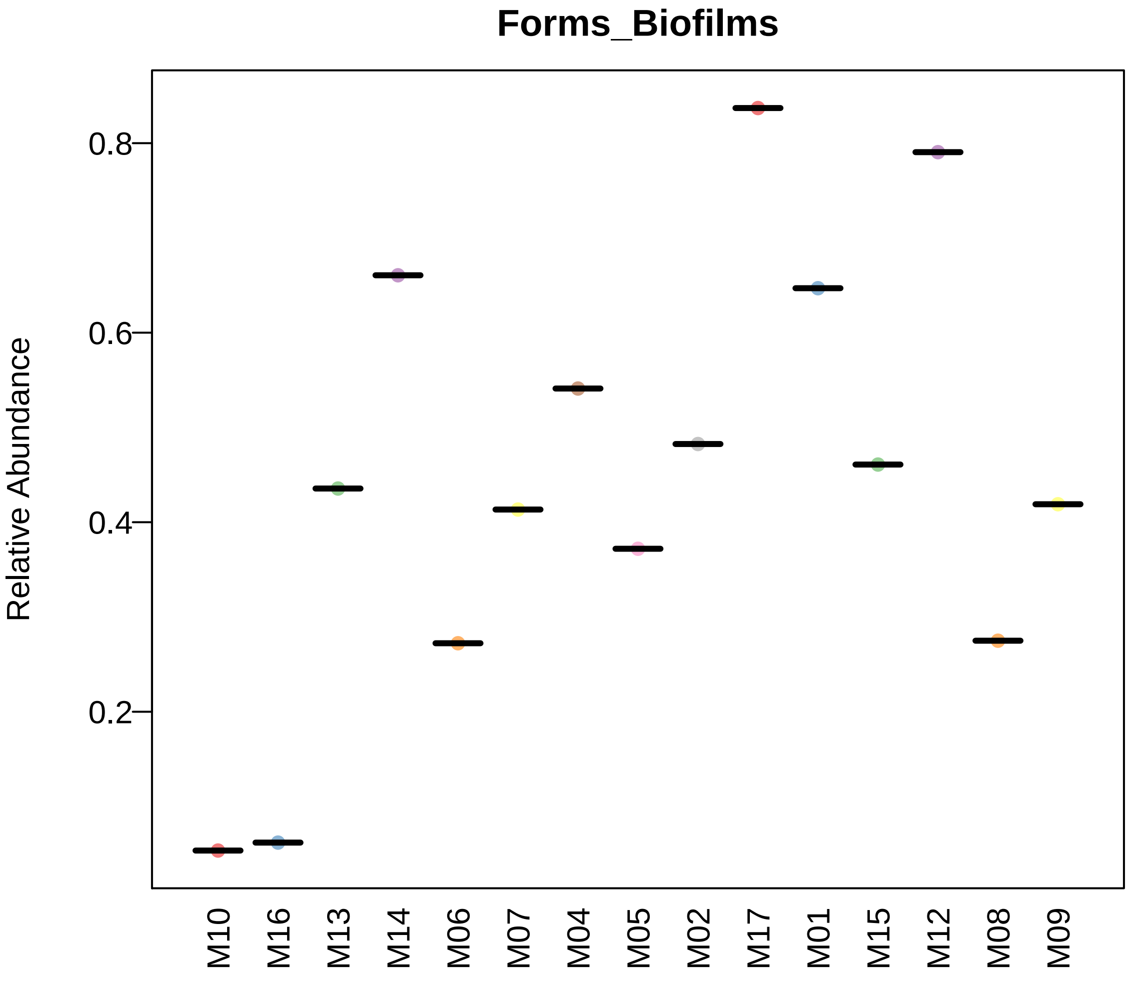
Forms_Biofilms
Relative Abundance
0.8
0.6
0.4
0.2
M10
M16
M13
M14
M06
M07
M04
M05
M02
M17
M01
M15
M12
M08
M09
